# Pregnancy outcome in more than 5000 births to women with viral hepatitis: a population-based cohort study in Sweden

**DOI:** 10.1007/s10654-017-0261-z

**Published:** 2017-05-26

**Authors:** Knut Stokkeland, Jonas Filip Ludvigsson, Rolf Hultcrantz, Anders Ekbom, Jonas Höijer, Matteo Bottai, Olof Stephansson

**Affiliations:** 1 Department of Medicine, Visby Hospital, St Görans Str. 8, 621 84 Visby, Sweden; 2 Department of Medicine, Gastroenterology and Hepatology Unit, Karolinska Institutet, Stockholm, Sweden; 3 Department of Pediatrics, Örebro University Hospital, Örebro, Sweden; 4 Department of Medical Epidemiology and Biostatistics, Karolinska Institutet, Stockholm, Sweden; 5 Division of Epidemiology and Public Health, School of Medicine, University of Nottingham, Nottingham, UK; 6 Division of Hepatology, Karolinska Hospital, Stockholm, Sweden; 7 Department of Medicine, Solna, Clinical Epidemiology Unit, Karolinska Hospital and Institutet, Stockholm, Sweden; 8 Unit of Biostatistics, IMM, Karolinska Institutet, Stockholm, Sweden; 9 Department of Women’s and Children’s Health, Karolinska Institutet, Stockholm, Sweden

**Keywords:** Preeclampsia, Preterm birth, Late neonatal death, Pregnancy outcomes

## Abstract

Previous studies have shown inconsistent results with respect to hepatitis B (HBV), hepatitis C (HCV) and pregnancy outcome. The aim of this study was to investigate pregnancy outcome in women with HBV or HCV. In a nationwide cohort of births between 2001 and 2011 we investigated the risks of adverse pregnancy outcomes in 2990 births to women with HBV and 2056 births to women with HCV using data from Swedish healthcare registries. Births to women without HBV (n = 1090 979), and births without HCV (n = 1091 913) served as population controls. Crude and adjusted relative risks (aRR) were calculated using Poisson regression analysis. Women with HCV were more likely to smoke (46.7 vs. 8.0%) and to have alcohol dependence (18.9 vs. 1.3%) compared with population controls. Most women with HBV were born in non-Nordic countries (91.9%). Maternal HCV was associated with a decreased risk of preeclampsia (aRR: 0.39, 95% CI: 0.24–0.64), but an increased risk of preterm birth (aRR: 1.32, 95% CI: 1.08–1.60) and late neonatal death (7–27 days: aRR: 3.79, 95% CI: 1.07–13.39) Preterm birth were also more common in mothers with HBV, aRR: 1.21 (95% CI: 1.02–1.45). Both HBV and HCV are risk factors for preterm birth, while HCV seems to be associated with a decreased risk for preeclampsia. Future studies should corroborate these findings.

**Electronic supplementary material:**

The online version of this article (doi:10.1007/s10654-017-0261-z) contains supplementary material, which is available to authorized users.

## Introduction

HBV and HCV affects 400 and 130–150 million people worldwide, respectively [1, 2]. In Sweden the incidence of HBV varies between 15 and 20 cases per 100 000 person-years, while the incidence of HCV has decreased to 20 per 100 000 person-years between 1997 and 2011 [3, 4]. Neither HBV nor HCV are cytopathogenic vira. Both elicit inflammatory responses in their hosts. Many women with HBV and HCV are in their fertile age and their pregnancies may be affected. Neither HBV or HCV passes the placental barriers but the inflammation the vira elicit may affect the pregnant women negative and result in negative pregnancy outcomes [5].

There are a few cohort and case–control studies that present negative pregnancy outcomes both in high- and low-endemic countries and regions in women with HBV and HCV. Both Reddick and Safir et al. found an excess risk of low birth weight in infants to HBV positive mothers, whereas no such increase was seen in four other studies [6–12]. Two studies have found a decreased risk for gestational hypertension and preeclampsia [9, 13], and there are also reports of excess risks of gestational diabetes and malformations in HBV [6, 7].

HCV has been linked to adverse pregnancy outcome, such as increased risk for gestational hypertension, preterm birth and low birth weight and small for gestational age birth [7, 14]. One study did not report an increased risk for preterm delivery [15].

The inconsistent findings on pregnancy outcome in patients with HBV and HCV motivated us to carry out a nationwide population-based study in a low-endemic country on adverse pregnancy outcome taking important confounding factors into account. The rationale for doing this in Sweden is the excellent health registers including the Birth Register and the free access to antenatal health care that Swedish women have assuring that we have included the vast majority of pregnancies in Sweden during the study period of our study. The aim of this study was to investigate pregnancy outcomes in women with HBV and HCV compared with population controls.

## Materials and methods

We used the Swedish Medical Birth Register (MBR) to identify deliveries between 2001 and 2011 [16]. The register contains prospectively collected demographic data, information on reproductive history, and complications that occur during pregnancy, delivery and the neonatal period.

The Swedish Patient Register (PAR) covers all public in-patient care in Sweden since 1987. Since 2001 information on out-patient hospital visits are included. Date of first diagnosis according to ICD-10 was recorded. We linked the women from the MBR to PAR using the unique personal identity number assigned to each citizen at birth or immigration [17]. We excluded twin pregnancies and women without personal identity numbers. From PAR we obtained information on women with a diagnosis of HBV and HCV according to the International Classification of Diseases, tenth revision (ICD-10). HBV was defined as ICD-10 codes B18.0 and B18.1 and HCV as B18.2 [18]. All births after the diagnosis of hepatitis B or C were included.

Maternal age at birth was classified into three groups: ≤24 years, 25–34 years and >35 years. We divided the births into two 5-year periods between 2001 and 2011. Cigarette smoking at first attendance to antenatal care was recorded as: no daily smoking, 1–10 cigarettes/day, and more than 10 cigarettes/day. Women were classified according to whether they were living with the infant’s father or not. Women were categorized as having body mass index (BMI) at first antenatal visit as 11.0–19.9 kg/m^2^, BMI 20.0–24.9 kg/m^2^, BMI 25.0–29.9 kg/m^2^ and BMI 30.0–60.0 kg/m^2^. Parity was divided into 1, 2, or 3 + deliveries. We classified women as having pre-pregnancy diabetes if they had a diabetes diagnosis in PAR prior to the first antenatal visit (ICD-10: E10 or E11). Alcohol dependence was defined as having a diagnosis of alcohol dependence (ICD-10: F10) and other dependencies were defined as having one of the dependence diagnoses (ICD-10: F11-19) before delivery.

Gestational diabetes (O24.4) and preeclampsia (O14 and O15) were defined according to ICD-10. Information on stillbirth at 28 weeks of gestation or later until 2008 and thereafter from gestational week 22 and onwards, and birth weight was obtained from the standardized pediatrics record, routinely filled out immediately after delivery. The Apgar score at 5 min after birth was categorized into: 0– 6 (“low Apgar score”), and between 7 and 10 (“normal”).

Low birth weight was defined as <2500 grams. Small for gestational age (SGA) birth was defined as birth weight <2 standard deviations (SD) below the mean for gestational age according to the sex-specific Swedish reference curve of estimated fetal growth [19]. Gestational age at birth was categorized into: very preterm (<32 weeks), moderately preterm (32–36 weeks), and term (≥37 weeks), and we even categorized the preterm into induced and spontaneous deliveries. Information on congenital malformations was obtained from discharge diagnosis at the delivery hospital (ICD-10: Q00-Q99). Information on infant mortality was obtained from the Causes of Death Register and categorized into early neonatal (0–6 days) and late neonatal (7–27 days) death. We excluded stillbirths in all analyses of pregnancy outcomes except for the analysis of stillbirth.

Information on the number of years of formal education completed as of 1 January 2012 was obtained from the Education Register and categorized as: <10 years, 10–12 years, or >12 years. Country of birth was categorized into: Sweden, other Nordic and non-Nordic. We defined high-epidemic countries for HBV as sub-Saharan Africa and East Asia, and high-epidemic countries for HCV as Northern Africa and Central Asia. The categorization of countries is presented in Supplementary Table 1.

Informed consent was not obtained as this was a register study and all information was anonymized. The study was approved by the local ethical committee in Stockholm, Sweden.

### Statistical method

To investigate the relationship between HBV and HCV and the binary outcomes of pregnancy and births, adjusted relative risks were calculated through Poisson regression with robust standard errors [20]. The ordinal outcome of gestational age was analyzed using ordinal logistic regression, resulting in odds ratios. The two nominal variables, induced and spontaneous deliveries, and Caesarean section was analyzed using multinomial logistic regression, resulting in relative risk ratios.

To estimate the association between HBV and HCV and the ordinal outcome of preterm birth, an ordered logistic proportional odds model was fitted, while Caesarean section was analyzed using multinomial logistic regression.

For the model concerning gestational diabetes, adjustments were made for maternal age, year of birth, parity, maternal smoking during pregnancy, if the mother lived with the father of the child, BMI, and maternal use of alcohol or other drugs, education and region of birth. For all other models, we also adjusted for diabetes mellitus. The variables age, year and BMI was treated as continuous. We also tested for interactions between hepatitis infections and addiction (alcohol or other dependencies). We even tested for other interactions and when significance was found we calculated aRR for births for women with HBV and for births for women with HCV. When the interaction effect was statistically significant, we estimated the aRR for the non-addicted group and the addicted group, separately and we included it in the model.

## Results

We included 1,093 969 singleton deliveries from the MBR. Of these, 2990 deliveries were to women with HBV and 2056 deliveries were to women with HCV. The population controls were women without HBV and HCV respectively. The maternal characteristics are presented in Tables 1 and 3.

**Table 1:** Descriptive data on women with Hepatitis B

	Hepatitis B 2990 (0.27%)	Non-hepatitis B 1,090,979 (99.73%)
Maternal age at delivery	*p* < 0.001	
≤24	506 (16.92)	156,906 (14.38)
25–34	1780 (59.53)	709,970 (64.91)
≥35	704 (23.55)	226,587 (20.70)
Calendar period of birth	*p* < 0.001	
2001–2006	1105 (36.96)	564,577 (51.75)
2007–2011	1885 (63.04)	527,038 (48.25)
Cigarette smoking	*p* = 0.014	
No	2623 (87.73)	955,048 (85.84)
1–10 cig./day	161 (5.38)	62,420 (5.72)
10+ cig./day	79 (2.64)	20 985 (1.92)
Missing	127 (4.25)	52 526 (4.81)
Parents living together	*p* < 0.001	
No	383 (12.81)	59,188 (5.43)
Yes	2484 (83.08)	981,125 (89.93)
Missing	123 (4.11)	50,666 (4.64)
BMI	*p* < 0.001	
11–19.9	379 (12.68)	96,045 (8.80)
20–24.9	1269 (42.44)	521,942 (47.84)
25–29.9	740 (24.75)	247,764 (22.71)
30–60	273 (9.13)	113,702 (10.42)
Missing	329 (11.00)	11,526 (11.22)
Parity	*p* < 0.001	
1	1014 (33.91)	488,887 (44.81)
2	989 (33.08)	396,034 (36.30)
3+	987(33.01)	206,058 (18.89)
Diabetes	*p* = 0.778	
No	2975 (99.50)	1,085,093 (99.46)
Yes	15 (0.50)	5886 (0.54)
Alcohol dependence	*p* < 0.001	
No	2934 (98.13)	1,077,030 (98.72)
Yes	56 (1.87)	13,949 (1.28)
Other dependencies	*p* < 0.001	
No	2930 (97.99)	1,079,104 (98.91)
Yes	60 (2.01)	11,875 (1.09)
Country of Birth	*p* < 0.001	
Sweden	232 (7.76)	868,673 (79.62)
Other Nordic	10 (0.33)	18,773 (1.72)
Others	2748 (91.91)	203,533 (18.66)
Educational level	*p* < 0.001	
≤9	1029 (34.41)	125,374 (11.49)
10–12	1004 (33.58)	44,984 (41.24)
13	762 (25.48)	499,411 (45.78)
Missing	195 (6.52)	16,310 (1.49)

The *p* value is from chi2-test

Compared with population controls, women with HBV were older (*p* < 0.001) and more often multipara (*p* < 0.001). In total 46.7% of women with HCV (*p* < 0.001) and 8.0% of women with HBV smoked during pregnancy (*p* = 0.004). The corresponding rates were 7.6% both in HBV and HCV controls.

In women with HCV there were 18.9% with a diagnosis of alcohol dependence compared to 1.3% of the controls (*p* < 0.001). Other dependencies were more frequent in women with HCV compared to control women (26.4 vs. 1%, *p* < 0.001). More than 90% of women with HBV were of non-Nordic origin.

In Table 2 we present pregnancy outcomes for women with HBV. We found a small increase in relative risk for preterm birth (aRR 1.21; 95% CI: 1.01–1.45). After adjustments, there were no significant increases in risk of preeclampsia, gestational diabetes or low Apgar score at 5 min in women with HBV. For elective Caesarean section there were an increased relative risk ratio (RRR) (aRRR 1.33; 95% CI: 1.12–1.59). The infants did not have significantly lower birth weight and were not more likely to be small for gestational age. We did find a significantly increased risk for late neonatal death (aRR 3.86; 95% CI: 1.09–13.70) in women with HBV compared to population controls (Table 3).

**Table 2:** Pregnancy outcomes for women with hepatitis B presented as relative risks

	Hepatitis B n = 2,990 (%)	Non-n = Hepatitis B 1,090,979(%)	Crude RR	Adjusted RR
Gestational diabetes	68 (2.23)	11,262 (1.03)	2.20 (1.69–2.87)	0.96 (0.71–1.27)
No	2922 (97.77)	1,079,717 (98.97)	Ref = 1.0	Ref = 1.0
Preeclampsia	59 (1.97)	30,030 (2.75)	0.72 (0.55–0.93))	0.90 (0.68–1.21)
No	2931 (98.03)	1,060,949 (97.25)	Ref = 1.0	Ref = 1.0
Caesarean section				
Acute	279 (9.40)	89,516 (8.23)	1.11 (0.97–1.28)	1.13 (0.96–1.32)
Elective	237 (7.99)	89,445 (8.22)	0.98 (0.84–1.13)	1.00 (0.84–1.17)
No	2 460 (82.61)	908,421 (83.55)	Ref = 1.0	Ref = 1.0
Apgar score at 5 min				
7–10	2 906 (97.94)	1,069,637 (98.37)	Ref = 1.0	Ref = 1.0
0–6	46 (1.58)	11,822 (1.09)	1.43 (1.06–1.91)	1.31 (0.93–1.84)
Data missing	15 (0.48)	5,923 (0.54)		
Low birth weight (<2500 g)	124 (4.18)	33,641 (3.10)	1.35 (1.13–1.62)	1.08 (0.88–1.33)
No	2,843 (95.82)	1,053,741 (96.90)	Ref = 1.0	Ref = 1.0
Small for gestational age	109 (3.67)	26,855 (2.47)	1.49 (1.22-1.81)	0.98 (0.78-1.22)
No	2,850 (96.06)	1 056,876 (97.17)	Ref = 1.0	Ref = 1.0
Data missing	8 (0.27)	3651 (0.36)		
Congenital malformation	101 (3.40)	38,469 (3.54)	0.96 (0.79–1.16)	0.99 (0.80–1.24)
No	2 866 (96.60)	1,048,913 (96.46)	Ref = 1.0	Ref = 1.0
Stillbirth	23 (0.77)	3 597 (0.33)	2.33 (1.55-3.50)	1.59 (0.96-2.59)
No	2 967 (99.23)	1 087 382 (99.67)	Ref = 1.0	Ref = 1.0
Early neonatal death (0-6)	4 (0.13)	1 210 (0.11)	1.21 (0.46-3.23)	1.09 (0.35-3.42)
No	2 963 (99.87)	1 086 172 (99.89)	Ref = 1.0	Ref = 1.0
Late neonatal death (7–27)	2 (0.07)	458 (0.04)	1.60 (0.40–6.42)	NPA
No	2965 (99.93)	1,086,924 (99.96)	Ref = 1.00	Ref = 1.00
Gestational age			1.21 (1.03–1.42)	1.22 (1.02–1.45)
Very preterm birth (<32 weeks)	27 (0.91)	7473 (0.69)		
Moderately preterm birth (32–36 weeks)	148 (4.99)	45,979 (4.23)		
Term births (37–44 weeks)	2791 (94.07)	1,033,321 (95.03)	Ref = 1.0	Ref = 1.0
Data missing	1 (0.03)	609 (0.06)		
Induced preterm	48 (1.6)	14,647 (1.3)	1.21(0.91–1.61)	1.28 (0.93–1–75)
Spontaneous preterm	127 (4.3)	38 805 (3.6)	1.21 (1.00–1.46)	1.20 (0.98–1.47)

Adjusted for mother’s age, year of birth, if the mother smokes at early pregnancy, if the mother lives together with the father of the child, BMI, parity, if the mother has diabetes mellitus, and if the mother is addicted to alcohol or are diagnosed with other dependencies. For gestational diabetes and preeclampisa stillbirths are included

The method used for measuring association between Caesarean section and liver disease was multinomial logistic regression. The interpretation of the estimates is in terms of relative rate ratios Adjusted for mother’s age, year of birth, if the mother smokes at early pregnancy, if the mother lives together with the father of the child, BMI, parity, if the mother has diabetes mellitus, and if the mother is addicted to alcohol or are diagnosed with other dependencies

*NPA* not possible to analyze

**Table 3:** Descriptive data on women with Hepatitis C

	HepC 2 056 (0.11%)	Non-hepC 1 091 913 (99.89%)
Maternal age at delivery	*p* < 0.001	
≤24	345 (16.78)	157,067 (14.38)
25–34	1168 (56.81)	708,802 (64.91)
≥35	543 (26.41)	226,044 (20.70)
Calendar period of birth	*p* < 0.001	
2001–2006	937 (45.57)	564,745 (51.72)
2007–2011	1119 (54.53)	527,168 (48.28)
Cigarette smoking	*p* < 0.001	
No	997 (48.49)	957,671 (87.61)
1–10 cig./day	533 (25.92)	62,048 (5.68)
10+ cig./day	427 (20.77)	20,637 (1.89)
Missing	99 (4.82)	52,544 (4.64)
Parents living together	*p* < 0.001	
No	538 (24.17)	59,033 (5.41)
Yes	1 404 (69.29)	982,205 (89.95)
Missing	114 (5.54)	50,675 (4.64)
BMI	*p* < 0.001	
11–19.9	155 (7.54)	96,269 (8.83)
20–24.9	864 (42.02)	522,347 (47.57)
25–29.9	521 (24.94)	247,983 (22.23)
30–60	229 (12.25)	113,746 (10.42)
Missing	287 (13.96)	111,568 (10.22)
Parity	*p* < 0.001	
1	805 (39.15)	489,096 (44.79)
2	681 (33.12)	396,342 (36.30)
3+	570 (27.72)	206,475 (18.91)
Diabetes	*p* = 0.005	
No	2040 (99.22)	1,086,028 (99.46)
Yes	16 (0.78)	5885 (0.54)
Alcohol dependence	*p* < 0.001	
No	1667(81.08)	1,078,297 (98.75)
Yes	389 (18.92)	13,616 (1.25)
Other dependencies	*p* < 0.001	
No	1513 (73.59)	1,080,521 (98.95)
Yes	543 (26.41)	11,398 (1.04)
Country of birth		
Sweden	1630 (79.43)	867,275 (79.43)
Other Nordic	50 (2.43)	18,733 (1.72)
Others	376 (18.29)	205,905 (18.86)
Educational level		
≤9	911 (44.31)	125,492 (11.49)
10–12	822 (39.98)	450,066 (41.22)
13	305 (14.83)	499,868 (45.78)
Missing	19 (0.88)	16,487 (1.51)

Preeclampsia was less common in HCV births (1.3%) than in the general population (2.8%), with an adjusted relative risk (aRR) of 0.39 (95% CI: 0.24–0.64). Some 21.6% of HCV women had a cesarean section, compared to 16.5% of controls. There was an increased risk for elective section, aRR 1.33 (95%CI: 1.12–1.59). There were 169 preterm births in women with HCV (8.2%) compared to 53,458 (5.3%) among the controls, corresponding to an aRR of 1.33 (95% CI: 1.12–1.59). There were 4 late neonatal deaths among women with HCV (0.20%), compared to 456 in controls (0.04%), aRR 3.79 (95% CI: 1.08–13.38), and there was an increased risk of induced preterm births, aRRR 1.89 (95% CI: 1.40–2.56) (Table 4). We did not find a decreased or increased risk for gestational diabetes or low 5 min Apgar score. The infants had no increased risk of being born with significantly lower birth weight, nor small for gestational age, and were not more likely to be stillborn or have malformations compared to controls.

**Table 4:** Pregnancy outcomes for women with hepatitis C: presented as relative risks

	Hepatitis C 2 056	Non-Hepatitis C 1 091 913	Crude RR	Adjusted RR
Gestational diabetes	28 (1.36)	11,302 (1.03)	1.31 (0.90–1.92)	1.18 (0.79–1.75)
No	2028 (98.64)	1,080,611 (98.97)	Ref = 1.0	Ref = 1.0
Preeclampsia	26 (1.27)	29,937 (2.75)	0.46 (0.31–0.69)	0.39 (0.24–0.64)
No	2018 (98.73)	1,058,368 (97.25)	Ref = 1.0	Ref = 1.0
Caesarean section				
Acute	185 (9.05)	89,601 (8.24)	1.17 (0.99–1.38)	1.09 (0.91–1.30)
Elective	256 (12.53)	89,426 (8.21)	1.62 (1.40-1.88)	1.33 (1.12–1.59)
No	1603 (78.42)	909,278 (83.55)	Ref = 1.0	Ref = 1.0
Apgar score at 5 min				
0–6	35 (1.71)	11,833 (1.09)	1.58 (1.13–2.21)	1.05 (0.68–1.61)
7–10	1991 (97.41)	1,070,552 (98.37)	Ref = 1.0	Ref = 1.0
Data missing	18 (0.88)	5920 (0.54)		
Low birth weight (<2500 g)	94 (4.60)	33,671 (3.10)	1.49 (1.20–1.83)	0.98 (0.77–1.22)
No	1950 (95.40)	1,054,634 (96.90)	Ref = 1.0	Ref = 1.0
Small for gestational age	79 (3.86)	26,885 (2.47)	1.57 (1.25–1.96)	0.98 (0.77–1.24)
No	1956 (95.69)	1,057,770 (97.19)	Ref = 1.0	Ref = 1.0
Data missing	9 (0.44)	3,650 (0.34)		
Congenital malformation	74 (3.62)	38,496 (3.54)	1.02 (0.82–1.28)	0.88 (0.67–1.15)
No	1970 (96.38)	1,049,809 (96.46)	Ref = 1.0	Ref = 1.0
Stillbirth	12 (0.58)	3608 (0.33)	1.76 (1.01–3.10)	1.20 (0.62–2.34)
No	2044 (99.42)	1,088,305 (99.67)	Ref = 1.0	Ref = 1.0
Early neonatal death (0–6)	2 (0.10)	1212 (0.11)	0.88 (0.21–3.51)	0.88 (0.21–3.58)
No	2042 (99.90)	1,087,093 (99.89)	Ref = 1.0	Ref = 1.0
Late neonatal death (7–27)	4 (0.20)	456 (0.04)	4.67 (1.75–12.46)	3.79 (1.08–13.38)
No	2040 (99.80)	1,087,849 (99.96)	Ref = 1.0	Ref = 1.0
Gestational age			1.75 (1.47–2.08)	1.32 (1.08–1.60)
Very preterm birth (<32 weeks)	27 (1.32)	7473 (0.69)		
Moderately preterm birth (32–36 weeks)	142 (6.95)	45,985 (4.22)		
Term births (37–44 weeks)	1 873 (91.63)	1,034,239 (95.03)	Ref = 1.0	Ref = 1.0
Data missing	2 (0.10)	608 (0.06)		
Induced preterm	67 (3.2)	14,628 (1.3)	2.53 (1.94–3.31)	1.89 (1.40–2.56)
Spontaneous preterm	102 (5.0)	38,830 (3.6)	1.45 (1.18–1.78)	1.09 (0.86–1.37)

Adjusted for mother’s age, year of birth, if the mother smokes at early pregnancy, if the mother lives together with the father of the child, BMI, parity, if the mother has diabetes mellitus, and if the mother is addicted to alcohol or are diagnosed with other dependencies. For gestational diabetes and preeclampisa stillbirths are included

The method used for measuring association between Caesarean section and liver disease was multinomial logistic regression. The interpretation of the estimates is in terms of relative rate ratios Adjusted for mother’s age, year of birth, if the mother smokes at early pregnancy, if the mother lives together with the father of the child, BMI, parity, if the mother has diabetes mellitus, and if the mother is addicted to alcohol or are diagnosed with other dependencies

*NPA* not possible to analyze

We tested for interaction between hepatitis B and alcohol dependence and hepatitis B and other dependencies but found no significant interactions. For hepatitis C and alcohol dependence we found an interaction regarding congenital malformations, aRR 1.46 (95%, CI 0.92–2.31) and without dependence, aRR 0.75, (95%, CI 0.54–1.03), *p* = 0.02. For hepatitis C and smoking we found an interaction only for congenital malformation. For smokers, aRR 1.21, (95%, CI 0.88–1.66) and for non-smokers, aRR 0.57, (95%, CI 0.88–1.66), *p* = 0.01. We found no interactions between hepatitis C and other dependencies. For hepatitis C and Nordic/non-Nordic births we found an interaction for Low Apgar score, aRR 0.83 (95%, CI 0.50–1.37) and non-Nordic, aRR 2.09 (95%, CI 0.98–4.45), *p* = 0.05. For hepatitis B and Nordic/non-Nordic births we found an interaction for congenital malformation, aRR 1.83, (95%, CI 1.04–3.22) and non-Nordic births, aRR 0.92 (95%, CI 0.98–1.16), *p* = 0.03.

We even evaluated whether a co-infection of the two hepatitis viruses had any additional effect on the pregnancy outcomes but found no such effects.

Restricting our data to women from high-endemic countries, HBV did not better predict adverse pregnancy outcomes, there were only an increased risk for induced preterm births, aRR1.70 (95% CI: 1.03–2.84) (there were only 20 births in women with HCV from high-endemic countries; Supplementary Table 2 and 3).

## Discussion

In this large nationwide study, we found an inverse relationship between HCV and preeclampsia risk, and a positive association with cesarean section, preterm birth and late neonatal death. HBV was associated with an increased risk for preterm birth.

The reduced prevalence of preeclampsia seen in HCV pregnancies in our study was confirmed in one previous study, whereas an increased risk of preeclampsia in HCV pregnancies has been reported by the majority of previous investigations [21–23]. The frequencies of preeclampsia found in our study are in line with results reported in other Nordic countries like Denmark [24]. Preeclampsia is a systemic syndrome that appears to originate in the placenta and is characterized by widespread maternal endothelial dysfunction. Implicit in this generalized endothelial dysfunction may be an imbalance of angiogenic and anti-angiogenic factors, with decreased levels of pro-angiogenic factors such as VEGF and Ang-2. [25, 26] Previous studies have reported elevated circulatory Ang-2 and VEGF levels in patients with hepatitis C [27, 28]. This could be a possible pathway for the findings of a decreased preeclampsia risk in our study.

Our data confirm earlier reports of an increased risk for Cesarean delivery in HCV pregnancies [7, 14, 21, 29]. Two studies have reported a reduced rate of cesarean delivery in women with HCV (8.6 vs. 12.7% in the control group corresponding to an odds ratio of 0.69 (0.53–0.88)) [6, 22]. Local or national recommendation regarding cesarean section may have affected our results, but use of cesarean section to prevent mother-to-child-transmission is not currently recommended in Sweden [30].

The increased risk of preterm birth could be attributed to HBV- and HBC-mediated inflammation, as chronic liver disease increase inflammatory cytokine production [31, 32]. Our findings are in line with earlier studies and a recent published meta-analysis on preterm births in women with HCV [33]. Previous studies of mothers with liver diseases and other chronic inflammatory diseases have shown increased risk of preterm deliveries [34–39]. Alcohol consumption could influence the increased risk of preterm birth in women with viral hepatitis. Even if we controlled for a more detailed information on alcohol dependence rather the crude information we have on hospitalization with a diagnosis of alcohol dependence, there may be residual confounding. Unregistered drug use, medication and uncontrolled socioeconomical factors may contribute. Several recent studies found alcohol to be a risk factor for preterm birth, and these results are in line with our findings [40–43]. Conversely, one study showed that women who consumed 2–3.5 drinks per week had a 20% reduction in risk of risk of preterm birth [44]. We have analyzed the interaction between alcohol and other drug dependences and we found a significant interaction in mothers with alcohol dependence and HCV infection. Increased risk of preterm deliveries may be caused by the alcohol use during pregnancy and the chronic inflammation caused by the HCV infection.

Almost half of women (47%) with HCV smoked at their first prenatal visit. This is a very high proportion compared with population controls [36]. Cigarette smoking may increase the risks of adverse pregnancy outcomes such as preterm birth and stillbirth [45].

There are several limitations in our study. We did not have information on medication, illicit drugs or alcohol that the woman may have used during pregnancy, as we only had information on previously reported dependence diagnoses. We did not have information on viremia in any period of the pregnancies, neither could we differ between acute or chronic viral infection and we did not have any antibody levels or lab-tests. We did not have information on life-style factors other than smoking, but adjusted for socioeconomic status and birth region If smokers among women with HCV and HBV were misclassified as non-smokers more often than controls, some of the reduced risk for preeclampsia could be explained because there are studies supporting an inverse relationship between smoking and preeclampsia [46]. We have tested for interactions between smoking and the hepatitis vira and we only found an interaction between HCV and congenital malformation. Although we adjusted for several potential confounders, we cannot rule out that residual confounding has influenced our risk estimates, Unregistered drug use, medication and uncontrolled socioeconomical factors may has influenced our risk estimates. Among the limitations is also our lack of data on monitoring and blood pressure control in antenatal care. Hence, if women with HCV were less likely to attend antenatal care (thereby not taking advantage of blood pressure medication) this could in part explain the reduced risk of preeclampsia.

The strengths of this study are many. The health registers in Sweden are of high quality. The PAR has a high positive predictive value while the MBR contains approximately 98% of all births in the study period [16]. In Sweden, prenatal care and delivery care are tax-funded, and the participation rate in the prenatal care program is almost 100%. The first prenatal care visit commonly takes place at the end of the first trimester. We examined a large birth cohort with more than 1 million births. Almost all births in the study period are included in our study and the risk estimates are likely representative for women with HBV and HCV. Similar studies are complicated to perform because of the relative rarity of the HBV and HCV infections in developed countries and the low number of included pregnancies. The MBR contains prospectively collected information both regarding the characteristics of women and information on the pregnancy outcomes. We were able to use all non-viral hepatitis births as controls. This strengthened our risk assessments.

In conclusion, both HBV and HCV are risk factors for preterm birth, while HCV seems to be associated with a reduced risk of preeclampsia. High quality prenatal and antenatal care is important for women with viral hepatitis and their infants even in countries with full access to antenatal health care.

## Electronic supplementary material

Below is the link to the electronic supplementary material.

Supplementary material 1 (DOCX 48 kb)

Supplementary material 2 (DOCX 26 kb)

## Abbreviations

RR: Relative risk
PAR: Swedish Patient Register
ICD: International classification of diseases
MBR: Swedish Medical Birth Register
BMI: Body mass index
SGA: Small for gestational age
CI: Confidence interval
