# Supplementary material for: Pregnancy outcome in more than 5000 births to women with viral hepatitis: a population-based cohort study in Sweden
Source: Eur J Epidemiol. 2017 May 26;32(7):617–25. doi: 10.1007/s10654-017-0261-z (PMC5570776; doi:10.1007/s10654-017-0261-z)
Supplement: Supplementary file 1 — Supplementary material 1 (DOCX 48 kb) [file 10654_2017_261_MOESM1_ESM.docx]

# Suppelementary table 1

High prevalence countries for hepatitis B:

Angola

Benin

Botswana

Burkina Faso

Burundi

Central african republic

Djibouti

Ekvatorial guinea

Ivory coast

Eritrea

Ethiopia

Gabon

Gambia

Ghana

Guinea

Guinea-bissau

Camerun

Kenya

Kongo

Democrati republic of congo

Kongo

Lesotho

Liberia

Malawi

Mali

Mauretania

Mocambique

Namibia

Niger

Nigeria

Rwanda

Senegal

Sierra Leone

Somalia

Swaziland

Sydafrika

Tanzania

Tchad

Togo

Uganda

Zambia

Zimbabwe

Hong Kong

Japan

China

North Korea

South Korea

Mongolia

Japan

High prevalence countries for hepatitis C

Kirgizistan

Turkmenistan

Kazakstan

Tadjikistan

Turkmenistan

Hong Kong

Japan

China

North Korea

South Korea

Mongoliet

Libya

Marocko

Sudan

Tunis

Algeria

Egypt
