# Supplementary material for: Pregnancy outcome in more than 5000 births to women with viral hepatitis: a population-based cohort study in Sweden
Source: Eur J Epidemiol. 2017 May 26;32(7):617–25. doi: 10.1007/s10654-017-0261-z (PMC5570776; doi:10.1007/s10654-017-0261-z)
Supplement: Supplementary file 2 — Supplementary material 2 (DOCX 26 kb) [file 10654_2017_261_MOESM2_ESM.docx]

**Suppelmentary Table 2:** Mothers with hepatitis B from high-endemic countries compared to mothers without hepatitis B from high-endemic countries (see Supplementary Table 1). The p value is from the Chi2 –test.

|  | **Hepatitis B**  **N=796**  **(3.00 %)** | **Non-hepatitis B**  **N=25 769**  **(97.00%)** |
| --- | --- | --- |
| **Maternal age at delivery** | **P=0.04** |  |
| ≤ 24 | 116 (14.57) | 4 235 (16.43) |
| 25-34 | 462 (58.04) | 15 420 (59.84) |
| ≥ 35 | 218 (27.39) | 6 114 (23.73) |
| **Calendar period of birth** | **P<0.001** |  |
| 2001-2006 | 277(34.80) | 10 642 (41.30) |
| 2007-2011 | 519 (65.20) | 15 127 (58.70) |
| **Cigarette smoking** | **P=0.20** |  |
| No | 753 (94.60) | 23 938 (92.89) |
| 1-10 cig./day | 9 (1.13) | 505 (1.96) |
| 10+ cig./day | 1 (0.13) | 83 (0.32) |
| Missing | 33 (4.15) | 1 243 (4.82) |
| **Parents living together** | **P=0.31** |  |
| No | 173 (21.73) | 5 828 (22.62) |
| Yes | 593 (74.50) | 18 7906 (72.59) |
| Missing | 30 (3.77) | 1 235 (4.79) |
| **BMI** | **P=0.50** |  |
| 11-19.9 | 90 (11.31) | 3 023 (11.73) |
| 20-24.9 | 271 (34.05) | 9 233 (35.83) |
| 25-29.9 | 221(27.76) | 6 484 (25.16) |
| 30-60 | 122 (15.33) | 3 849 (14.94) |
| Missing | 92 (11.56) | 3 180 (12.34) |
| **Parity** | **P<0.001** |  |
| 1 | 199 (25.00) | 8 130 (31.55) |
| 2 | 218 (27.39) | 7 180(27.86) |
| 3+ | 379 (47.61) | 10 459 (40.59) |
| **Diabetes** | **P=0.777** |  |
| No | 792 (99.50) | 25 644 (99.51) |
| Yes | 4 (0.50) | 125 (0.42) |
| **Alcohol dependence** | **P=0.85** |  |
| No | 793 (99.62) | 25 682 (99.66) |
| Yes | 3 (0.38) | 87 (0.49) |
| **Other dependencies** | **P=0.32** |  |
| No | 795 (99.87) | 25 685 (99.67) |
| Yes | 1 (0.13) | 84 (0.33) |
| **Educational level (years)** | P<0.01 |  |
| -9 | 321 (40.33) | 9 762 (37.88) |
| 10-12 | 215 (27.01) | 7 201 (27.94) |
| 13- | 154 (19.35) | 6 073 (23.57) |
| Missing | 106 (13.32) | 2 733 (10.61) |

**Suppelementary table 3**.**Pregnancy outcomes for women with hepatitis B** from high-endemic countries: presented as relative risks presented as relative risks. Adjusted for mother’s age, year of birth, if the mother smokes at early pregnancy, if the mother lives together with the father of the child, BMI, parity, if the mother has diabetes mellitus, and if the mother is addicted to alcohol or are diagnosed with other dependencies. For gestational diabetes and preeclampisa stillbirths are included.

The method used for measuring association between Caesarean section and liver disease was multinomial logistic regression. The interpretation of the estimates is in terms of relative rate ratios Adjusted for mother’s age, year of birth, if the mother smokes at early pregnancy, if the mother lives together with the father of the child, BMI, parity, if the mother has diabetes mellitus, and if the mother is addicted to alcohol or are diagnosed with other dependencies. NPA=not possible to analyze.

|  | **Hepatitis B**  **N=796** | **Non-Hepatitis B**  **N= 25 769** | **Crude RR** | **Adjusted RR** |
| --- | --- | --- | --- | --- |
| **Gestational diabetes** | 24 (3.0) | 701 (2.7) | 1.11 (0.66-1.86) | 1.00 (0.58-1.72) |
| No | 772 (97.0) | 25 068 (97.3) | Ref=1.0 | Ref=1.0 |
| **Preeclampsia** | 23 (2.9) | 686 (2.7) | 1.09 (0.69-1.72) | 0.87 (0.50-1.49) |
| No | 773 (97.1) | 31 780 (97.3) | Ref=1.0 | Ref=1.0 |
| **Caesarean section** | 164 (20.9) | 4 990 (19.5) | 1.07 (0.90-1.27) | 1.15 (0.95-1.38) |
| Acute | 88 (11.2) | 2 741(10.7) | 1.06 (0.82-1.49) | 1.22 0.91-1.65) |
| Elective | 76 (9.7) | 2 249 (8.8) | 1.11 (0.82-1.37) | 1.16 (0.84-1.59) |
| No | 622 (79.1) | 20 596 (80.5) | Ref=1.0 | Ref=1.0 |
| **Apgar score at 5 minutes** |  |  | 1.61 (1.06-2.44) | 1.34 (0.78-2.32) |
| 0-6 | 22 | 447 |  |  |
| 7-10 | 756 | 324 962 | Ref=1.0 | Ref=1.0 |
| Data missing | 8 | 177 |  |  |
| **Low birth weight(<2500 g)** | 33 (4.2) | 1 035 (4.1) | 1.04 (0.74-1.46) | 0.93 (0.60-1.45) |
| No | 753 (95.8) | 24 551 (95.9) | Ref=1.0 | Ref=1.0 |

|  | **Hepatitis B**  **N=786** | **(Non-Hepatitis B**  **N= 25 586** | **Crude RR** | **Adjusted RR** |
| --- | --- | --- | --- | --- |
| **Small for gestational age** | 43 (5.5) | 1 323 (5.2) | 1.06 (0.79-1.42) | 0.98 (0.67-1.42) |
| No | 741 (94.2) | **25 187 (94.5)** | Ref=1.0 | Ref=1.0 |
| missing | 2 (0.3) | **76 (0.3)** |  |  |
| **Congenital malformation** | 30 (3.8) | 856 (3.3) | 1.14 (0.80-1.63) | 1.11 (0.73-1.68) |
| No | 756 (96.2) | 24 730 (96.7) | Ref=1.0 | Ref=1.0 |
| **Stillbirth** | 10 (1.3) | 183 (0.7) | 1.77 (0.94-3.23) | 1.70 (0.80-3.60) |
|  | 786 (98.7) | **25 586 (99.3)** | Ref=1.0 | Ref=1.0 |
| **Early neonatal death (0-6)** | 2 (0.2) | 39 (0.1) | 1.67 (0.41-6.88) | 1.51 (0.21-11.88) |
| No | **784 (99.8)** | **25 547 (99.9)** | Ref=1.0 | Ref=1.0 |
| **Late neonatal death (7-27)** | 0 (0) | 15 (<0.1) | NPA | NPA |
| No | 786 (100.0) | 25 571 (99.9) | Ref=1.0 | Ref=1.0 |
| **Gestational age**1 |  |  | 1.29 (0.94-1.77) | 1.34 (0.93-1.92) |
| Very preterm birth (<32 weeks) | 6 (0.8) | 265 (1.0) |  |  |
| Moderately preterm birth (32-36 weeks) | 39 (5.0) | 877 (3.4) |  |  |
| Term births (37-44 weeks) | 741 (94.3) | 31 028 (95.1) | Ref=1.0 | Ref=1.0 |
| Data missing | 0 (0) | 17 (0.1) |  |  |
| Induced | 27 (3.4) | 423 (1.7) | 1.40 (0.87-2.25) | 1.70 (1.03-2.84) |
| Spontaneous | 18 (2.3) | 719 (2.8) | 1.24 (0.81-1.87) | 1.12 (0.69-1.82) |
